# Graph pyramids for protein function prediction

**DOI:** 10.1186/1755-8794-8-S2-S12

**Published:** 2015-05-29

**Authors:** Tushar Sandhan, Youngjun Yoo, Jin Young Choi, Sun Kim

**Affiliations:** 1 Perception and Intelligence Lab, Department of Electrical and Computer Engineering, Seoul National University, 151-742 Seoul, South Korea; 2 Department of Computer Science and Engineering, and The Bioinformatics Institute, Seoul National University, 151-742 Seoul, South Korea

**Keywords:** Protein classification, Protein homology, Protein-Protein similarity network, Network biology

## Abstract

**Background:**

Uncovering the hidden organizational characteristics and regularities among biological sequences is the key issue for detailed understanding of an underlying biological phenomenon. Thus pattern recognition from nucleic acid sequences is an important affair for protein function prediction. As proteins from the same family exhibit similar characteristics, homology based approaches predict protein functions via protein classification. But conventional classification approaches mostly rely on the global features by considering only strong protein similarity matches. This leads to significant loss of prediction accuracy.

**Methods:**

Here we construct the Protein-Protein Similarity (PPS) network, which captures the subtle properties of protein families. The proposed method considers the local as well as the global features, by examining the interactions among 'weakly interacting proteins' in the PPS network and by using hierarchical graph analysis via the graph pyramid. Different underlying properties of the protein families are uncovered by operating the proposed graph based features at various pyramid levels.

**Results:**

Experimental results on benchmark data sets show that the proposed hierarchical voting algorithm using graph pyramid helps to improve computational efficiency as well the protein classification accuracy. Quantitatively, among 14,086 test sequences, on an average the proposed method misclassified only 21.1 sequences whereas baseline BLAST score based global feature matching method misclassified 362.9 sequences. With each correctly classified test sequence, the fast incremental learning ability of the proposed method further enhances the training model. Thus it has achieved more than 96% protein classification accuracy using only 20% per class training data.

## Background

The life of an organism is encrypted in the sequence of a genome, but decryption of the genetic information depends upon functions of the proteins that it encodes. The assignment of biological or biochemical roles to proteins has many challenges. Knowing just amino-acid sequence and structure of a protein does not guarantee that we can predict everything about that protein. However these measures are a good starting point for quickly predicting protein functions with the help of known homology. There are plenty of proteins which have totally unknown functions and the whole genome sequencing projects are major sources of these. So an approach based on protein homology is a fast, approximate and a primary way used to tackle such a daunting task of protein function prediction. The rationale behind this is that two proteins with similar sequence or structure could evolve from a common ancestor and thus have similar functions.

The homology of protein sequence is usually found by assessing similarity between pairs of sequences. An optimal algorithm based on dynamic programming like Needleman-Wunsch [1] is computationally inefficient for searching similar sequences in the large protein database. So most of the existing methods use suboptimal algorithms like BLAST [2] for matching a pair of sequences. Searching for only the highest scoring match in a protein database is nothing but looking for the global feature in the sequence similarity space.

**Figure 1:**
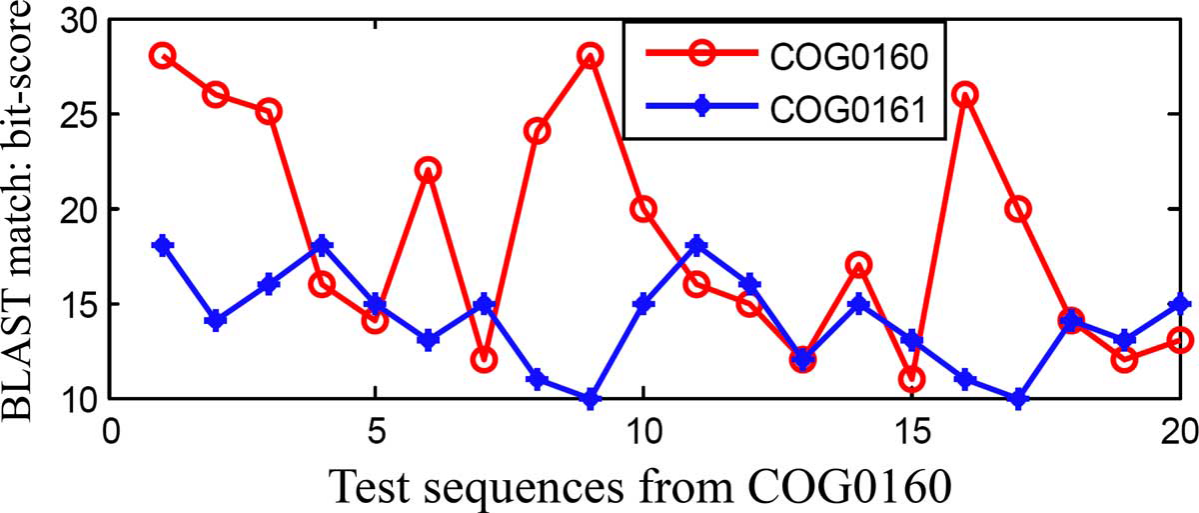
**Limitations of the global feature (the highest BLAST match score)**. Maximum BLAST matching scores of the test sequences from COG0160 protein family [6] with themselves and with another COG0161 protein family. It shows that the classification among closely related protein families, based only on the global feature becomes complicated.

Global features try to succinctly summarize the raw data, so they are rich in semantics. They have been found to be useful in the domains where semantic analysis of the raw data is important for pattern recognition, like audio event recognition [3] and video analysis [4,5]. But the sequences of related proteins can diverge beyond the point where their relationship becomes hard to be detected by such a global feature based methods. Figure 1 shows the protein classification result based on only global features. A couple of protein families are chosen for testing from the Cluster of Orthologous Groups of proteins (COG) database [6] and only 20 sequences from the COG0160 family are separated for evaluation. Some sequences from the COG0160 also show high (BLAST [2] matching) bit scores with the COG0161 family, instead of with their own protein family. So when the protein families are closely related, protein classification based only on global features becomes difficult and erroneous.

In attempts to overcome the above limitations, various matching methods have been developed that use the features extracted from multiple sequence alignment (MSA) of the protein family sequences. These methods use sequence templates [7] and profiles of the sequences [8] as features. They ask for accurate MSA of related sequences with low residue identities which requires some domain expertise. These profile based methods use ad hoc scoring systems without associating any evolutionary meaning to it [9], unlike PAM or BLOSUM [10]. These factors put limitations over these methods for using them for large protein databases.

Pattern recognition based only on local features could be useful for analyzing the large amount of data in real time like abnormal event detection from video [11]. Use of only local features is a trade-off between speed and the accuracy. Domains like biometrics, as well as bioinformatics, require high recognition accuracy as well as reliability. So methods which fuse local and global features have improved recognition performance [12].

MSA of a protein family reveals selective pressures for conservation of specific residues with evolutionary functional importance. Some MSA regions seem to tolerate insertions and deletions while others tend to remain conserved. So position-specific features from MSA are desirable when searching databases for homologies. Profile HMM, a generative model used widely for protein sequence classification, uses these position-specific features [13]. It uses global as well as local features by considering multiple sequences at the same time. But it lacks quick and incremental training functionality. After classification of a test sequence by profile HMM, to update the training model, MSA need to be calculated again and after that the new model parameters will be estimated from it. MSA is a time consuming process and also limits the number of sequences to be used for sequentially updating the training model. Unlike profile HMM, in the proposed graph based method, incremental training is performed easily and quickly. Test sequences can be easily added, either sequentially or all at a time, to the original graph to produce the new trained graph. This puts no limit on the number of test sequences to be added for updating the training model. In fact, the more new correct sequences added, the better the model will become.

Methods based on similarity clustering, k-nearest neighbors, phylogenetic clustering [14], gene fusion analysis [15] look for closely interacting sequences near the query sequence. They fail to account for interactions among the closely interacting neighborhood. Thus it leaves room for further performance improvement.

Intermediate sequence search (ISS) has also been successfully used for detecting remote homology [16]. For the sequences whose homology cannot be established by a direct comparison, ISS attempts to relate them through a third weakly interacting sequence with them. Thus for detection of remotely related protein sequences, use of intermediate sequences has been known to increase the predictive power significantly [17]. However, use of intermediate sequences can propagate errors dramatically when they are not of the same function. Excessive inclusion of the false positives can be effectively controlled by using graph theory. For example, Kim and Lee [18] used biconnectedness and articulation points to control the false positives effectively in an iterative manner. However, the relationships among sequences become very complicated as the number of sequences increases, so these relationships should be defined at multiple levels in a systematic manner. Thus our graph pyramid approach is an important solution for tackling above issues. The proposed method tightly controls false positives by considering strong interactions (global features) as well as all weak interactions (local features) in the graph with a hierarchical manner.

Protein-Protein Interaction (PPI) [19] plays a critical role in many biological processes. Protein expresses its functions when it interacts with the other proteins [20]. So PPI is a vital information for protein function prediction. On the other hand, understanding protein functions is critical for understanding the various biological processes [21]. PPI is modeled as a network, with protein sequences represent the nodes and biological protein interactions depict the edges in the network [19]. Protein function prediction methods based on PPI are promising as well as producing high performance but the availability of high throughput PPI data is an essential requirement for them [20,21]. So we propose a graph based protein classification method, which requires only amino-acid protein sequences as an input data. An edge in the graph is constructed by using the protein sequence similarity measure instead of PPI to produce the new Protein-Protein Similarity (PPS) network.

### Motivation and contributions

Relationships among biological sequences can be effectively represented by building a PPS network. However, in protein families, modularity, local clustering and scale-free topology coexist [19]. Thus use of single graph for modeling them has not been efficient. Here we propose the graph pyramid approach, where multiple graph features are used at different levels for modeling protein families. Along with this, the proposed algorithm using hierarchical voting scheme, tries to blend important characteristics from the PPI network and ISS methods for protein classification. This makes it possible to more objectively and reasonably predict the protein functions with high accuracy.

**Figure 2:**
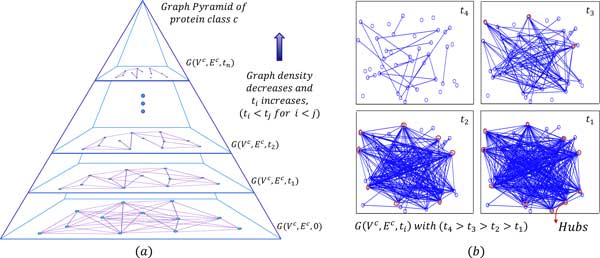
**The Graph Pyramid (GP)**. (*a*) Graph pyramid: a hierarchical analysis of the graph, (*b*) Building of the Protein-Protein similarity (PPS) network with varying threshold, shows the formation of hubs. Here class *c *is formed by some randomly chosen proteins from COG0160 [6], which consists of 4-aminobutyrate and related aminotransferases.

An edge connecting a pair of nodes with the largest weight in the PPS network represents the strongest match and it accounts for the global feature in similarity space. Only considering the nodes connected through the edges having lower weights, constitutes weak local features and with also considering interactions among weakly interacting nodes boost up the local features. In addition, PPS networks are analyzed hierarchically in the form of the Graph Pyramid (GP). It helps to extract more vital information about proteins from the network topology and looks for stronger global and local features. Unlike most of the existing methods, the proposed approach also shows that 'how the query sequence interacts with each protein in the family'. PPS interaction topology shows 'small world network' [19] properties like the PPI network (see Figure 2(b)). This helps and guides in devising important graph features (discussed in section 'Graph structured features').

This paper is organized as follows. In the section 'Methods', we present the PPS network construction approach and its modeling via graph pyramid. The same section also discusses the important graph topological or graph structured features and the hierarchical protein classification algorithm. Experimental evaluation results of the proposed method, their comparison with existing prediction methods and the techniques of searching for the optimal algorithm parameters are presented in section 'Results'. We conclude our work with discussions about related issues and the direction of our future work.

## Methods

Similarity between sequences can be assessed by local or global alignment score. A score is a numerical value that describes the overall quality of an alignment. The protein sequence classification algorithm which relies solely on an optimal alignment score is not practical for a large protein database. Protein sequence similarity can be readily found by suboptimal alignment using protein-protein BLAST [2]. Bitscore is a rescaled version of the raw alignment score that is independent of the size of the search space. P-value is the probability that the match is random. For multiple testing, P-value is corrected by multiplying it with size of the search space to get the E-value. The lower the Evalue, the more significant the score is. Thus E-values and Bit-scores carry slightly different information. So there should be an unified measure which combines the properties of both. To do this, the EB-score between sequences *s*_1 _and *s*_2 _is defined as follows,

(1) $$\begin{array}{c} eb=-log\left(\text{E}-\text{value}\left(s_{1},s_{2}\right)\right)\times \text{Bit-score}\left(s_{1},s_{2}\right)\\ \text{EB-score}\left(s_{1},s_{2}\right)=eb\times H\left(eb\right), \end{array}$$

where *H*(*·*) is the Heaviside-step function,

(2) $$H(x)=\{\begin{array}{ll} 1 & \text{if }x\geq 0\\ 0 & \text{otherwise}. \end{array}$$

The performance comparison given in the section 'Results', shows importance of the EB-score over the Bitscore.

### Graph construction

The proposed algorithm uses the graphical modeling of protein sequences from each protein family/class. Consider the large protein database having *M *classes. Let the set of class labels be given as ${\mathbb{C}}_{M}=\left\{c_{1},\;c_{2},\cdots \;,c_{M}\right\}$. Consider one of the protein classes, $c\in {\mathbb{C}}_{M}$, then let $s_{t_{i}}^{c}$ be the *i^th ^*training sequence in that class. Similarly $s_{q}^{c_{q}}$ is the new query or test sequence and its class label *c_q _*is what we have to find.

Let the protein family or the class *c *has *N *training sequences (*N *may be different for different $c\in {\mathbb{C}}_{M}$), then the set of vertices for class *c *is defined in the similarity space as, $V^{c}=\left\{s_{t_{1}}^{c},s_{t_{2}}^{c},\cdots s_{t_{N}}^{c}\right\}$. Strength of an edge between the vertices and is given as,

(3) $$e_{i,j}^{c}=\left\{\begin{array}{cc} 0 & \text{if}\;i=j\\ \text{EB-score}\left(s_{t_{i}}^{c},s_{t_{j}}^{c}\right) & \text{otherwise} \end{array}\right)$$

These edges form a set $E^{c}=\left\{e_{1,1}^{c},e_{1,2}^{e},\cdots \;,e_{N,N}^{c}\right\}$. Now the graph of a protein class $c\in {\mathbb{C}}_{M}$ is given by *G*(*V^c^, E^c^*). This is a weighted and an undirected graph. An edge weight is nothing but the strength of protein sequence similarity.

To construct the graph of each protein class, we just need to consider interaction (EB-score) among all protein sequences within that class. Number of proteins in a class is far smaller than that of the entire database. So graphs of all protein classes can be easily and independently constructed by using protein-protein BLAST within the corresponding classes.

### Graph analysis

In protein similarity graphs, modularity, local clustering and scale-free topology [19] coexist. To explain this phenomenon we need the hierarchy, so graphs are analyzed in the hierarchical manner. At each hierarchical level, the edges with weights lower than a certain threshold are pruned. Now surviving edges are considered to be weightless. So the graph structure changes along hierarchical levels, as well as the graph becomes unweighted and remains undirected. This hierarchical analysis helps to extract different graph features for weakly similar hits (sequence matches) and thus captures the complex relationship between sequence similarity and protein function.

For any set  S, consider c∈S, and *i *as an indicator variable, and let ∅ be the null (empty) set, ∅ = {} then 'set element' is formed as

(4) $$\delta \left(c,i\right)=\left\{\begin{array}{cc} \left\{c\right\} & \text{if}\;i=1\\ 0̸ & \text{otherwise}. \end{array}\right)$$

Cardinality of a set (|S|), is the number of elements present in it. For the graph of *c*, at certain hierarchy (i.e. at threshold *t*), the edge set is given as,

(5) $$E_{t}^{c}=\underset{e_{i,j}^{c}\in E^{c}}{⋃}\delta \left(e_{i,j}^{c},H\left(e_{i,j}^{c}-t\right)\right).$$

The corresponding graph is given as $G\left(V^{c},E_{t}^{c}\right)$. For notation simplicity lets represent it as *G*(*V^c^, E^c^, t*), instead of $G\left(V^{c},E_{t}^{c}\right)$ and note that *G*(*V^c^, E^c^*) = *G*(*V^c^, E^c^*, 0).

For any sets $S_{1}$ and $S_{2}$, define the set addition function (like a multiset sum) as,

(6) $$S_{1}⊎S_{2}=\left\{S_{1}\cup S_{2},S_{1}\cap S_{2}\right\}$$

and note that $\left|S_{1}⊎S_{2}|=|S_{1}+S_{2}\right|$, thus added set contains repetitive elements when $S_{1}\cap S_{2}\neq 0̸$. For example, let $S_{1}=\left\{c_{1},c_{2}\right\}$ and $S_{2}=\left\{c_{2},c_{3}\right\}$ then $S_{1}⊎S_{2}=\left\{c_{1},c_{2},c_{3},c_{2}\right\}$. This operation obeys the associative and the commutative laws like numerical addition. As defined earlier, $s_{q}^{c_{q}}$ is the query sequence from the unknown class *c_q_*. Now $V_{q}^{c}=V^{c}⊎s_{q}^{c_{q}}$, and edges among the vertices in the set is given by an edge set $E_{q}^{c}$. After adding $s^{c_{q}}$ to the original graph *G*(*V^c^, E^c^*), we will get the new graph $G\left(V_{q}^{c},E_{q}^{c}\right)$.

### Graph Structured Features (GSF)

Most of the real world and biological (scale-free) networks communicate via a few highly connected nodes known as Hubs. These hubs determine the properties of networks [19]. In real world networks like airline route maps, the important cities form hubs. Proteins with high degrees of connectedness are more likely to be essential for survival than proteins with lesser degrees [22]. Gene duplication leads to growth and preferential attachment in biological networks [23]. This leads to translating the proteins having high similarity. This shows the possibility of hub formation in the protein family graphs, *G*(*V^c^, E^c^*), in the similarity space.

Figure 2(b) shows building of the PPS network with a varying threshold for one of the COG [6] protein families. We can see that as the threshold is lowered, trivially more edges are formed but most of them are associated with only particular nodes (hubs). Thus hubs are getting stronger and becoming more evident in the PPS network. We are not interested in the detailed assessment of whether the network is scale-free (a power-law degree distribution [19]) or not. But the above analysis helps to guide us for finding proper features which take graph structure (i.e. complex relationships among the protein sequences) into account. Also, the different protein families have different characteristics. Thus use of a single graph feature may not be effective. Features are selected such that they could extract different but vital network information.

#### Average Clustering coefficient (AC)

For a node *n*, the clustering coefficient *C_n_*, measures the extent to which neighbors of *n *are also the neighbors of each other [19]. Thus it is nothing but the density of sub-graph induced by the neighborhood of *n*. Consider the graph *G*(*V, E*) with *n *∈ *V *. Let ℕ*_n _*be the number of neighbors of *n *and $E_{n}$ be the number of edges between them, then the *AC *is given by,

(7) $$AC\left(G\left(V,E\right)\right)=\frac{1}{\left|V\right|}\underset{n\in V}{\sum }\left(\frac{2E_{n}}{{\mathbb{N}}_{n}\left({\mathbb{N}}_{n}-1\right)}\right).$$

**Figure 3:**
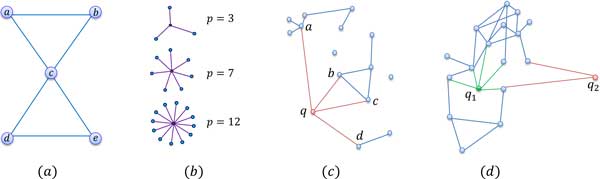
**Illustrations for graph structured features**. (*a*) Exemplar graph to explain the features average clustering coefficient (*AC*) and rich club coefficient (*RC*); (*b*) star motifs *SM *with different powers; (*c*) Illustration to explain that the number of triangles (*TR*) and the *SM *capture different network properties; (*d*) Different queries affect graph spread (graph energy *GE*) differently.

A clique is a maximal complete sub-graph where all the vertices are connected. *C_n _*quantifies how close the neighbors of a node are, to form a clique among themselves. It represents the potential modularity of a network and *C_n _*of the most real networks is much larger than that of a random networks. *AC *distribution is found to be effective for an identification of a modular organization of the metabolic networks [24]. Consider the example shown in Figure 3(a). Node *c *has 4 neighbors $\left({\mathbb{N}}_{c}\right)$, having 2 connected edges $\left(E_{c}\right)$ among them (*a *to *b *and *d *to *e*), which forms $C_{c}=\frac{1}{3}$ and then $AC=\frac{13}{15}$. When $s_{q}^{c_{q}}$ is attached to *G*(*V^c^, E^c^*), it may change its *AC*. For a given the change in *AC *is given as,

(8) $$\text{Δ}AC\left(c,t\right)=AC\left(G\left(V_{q}^{c},E_{q}^{c},t\right)\right)-AC\left(G\left(V^{c},E^{c},t\right)\right).$$

#### Rich Club coefficient (RC)

The 'rich-club' phenomenon refers to the tendency of nodes with high centrality to form tightly interconnected communities. Degree (*d*) of a node is the number of directly connected neighbors. High degree nodes (rich nodes) are much more likely to form tight and well interconnected sub-graphs than low degree nodes [25]. Thus hubs are generated through 'rich-gets-richer' mechanism. A quantitative definition of the rich-club phenomenon is given by the rich-club coefficient (*φ*). Let ${\mathbb{N}}_{d>r}$ be the number of vertices having degree greater than *r *and $E_{d>r}$ be the number of edges among those vertices then,

(9) $$RC\left(G\left(V,E\right),r\right)=\phi \left(r\right)=\frac{2E_{d>r}}{{\mathbb{N}}_{d>r}\left({\mathbb{N}}_{d>r}-1\right)}.$$

For the example shown in Figure 3(a), $\phi \left(1\right)=\frac{3}{5}$. In a complex network, *φ *is a novel probe for finding topological correlations and it yields vital information about the underlying architecture of the network [25]. Similarly as explained earlier, the change in *RC *is given as,

(10) $$\begin{array}{c} \text{Δ}RC\left(c,t,r\right)=RC\left(G\left(V_{q}^{c},E_{q}^{c},t\right),r\right)-\\ \;\;\;\;\;\;\;RC\left(G\left(V^{c},E^{c},t\right),r\right). \end{array}$$

#### Star Motifs (SM)

Previous analysis showed the existence of hubs in the PPS network. Features like mean path length and degree distribution [19], nicely quantifies hub like properties of the network. But mean path length is computationally very expensive for large protein database, so simple and elegant features are needed. Hubs indicate existence of star shaped patterns (*star-motifs *). Let power (*p*) of *SM *be the degree of the central (center of the 'star' pattern) node. Figure 3(b) shows the *SM *with various powers. Each node in the training graph is already assigned with the node degree, so that the *SM *can be easily computed. This simple feature answers the questions like, 'does the $s_{q}^{c_{q}}$ give rise to the new hubs' and 'what is the increased strength of the hubs'. Let *SM*(*·, p*) finds the number of *SM *of power *p *then change in SM is given as,

(11) $$\begin{array}{c} \text{Δ}SM\left(c,t,p\right)=SM\left(G\left(V_{q}^{c},E_{q}^{c},t\right),p\right)-\\ \;\;\;\;\;\;\;SM\left(G\left(V^{c},E^{c},t\right),p\right). \end{array}$$

#### Triangles (TR)

In a graph, the smallest clique with three nodes is a triangle. The number of triangles gives an important information about structure of the PPS network. Let the graph *G*(*V^c^, E^c^*) be represented by *N × N *adjacency matrix [*E^c^*], whose (*i, j*)*^th ^*entry is given as, $\left[E^{c}\right]\left(i,\;j\right)=H\left(e_{i,j}^{c}\right)$, then the number of triangles in the graph are given as,

(12) $$TR(G\left(V^{c},E^{c})\right)=\frac{1}{6}\overset{N}{\underset{i=1}{\sum }}{\left[E^{c}\right]}^{3}\left(i,i\right).$$

Either the graph is dense or sparse, TR can be computed readily within the same time as the computation is only associated with the matrix of the same size. TR inherently captures different network properties than SM. Figure 3(c) illustrates this difference more elaborately. Query node *q *interacts with the nodes *a, b, c *and *d*. So they constitute an 'interacting neighborhood' to the query. TR has capability to simultaneously assess the interactions within interacting neighborhood. After the query interaction, one extra triangle is formed since only the nodes *b *and *c *were previously interacting. Whereas at node *a SM *power has increased from 3 to 4. Formation of new triangles in a graph indicates the fact that 'query interacts simultaneously to the already interacting nodes'. Newly formed number of triangles due to query interaction Δ*TR*(*c, t*), are found similarly as equation (8).

#### Graph Energy (GE)

The original graph structure changes when the query interacts with it. Figure 3(d) shows two different type of query interaction with the same graph, where an edge length is proportional to its weight. When only *q*_1 _interacts with a graph then its spread remains almost unaffected but in case of *q*_2 _interaction, the spread of a graph alters drastically. *GE *is defined as the sum of the absolute values of the eigenvalues of the adjacency matrix [26], and given as follows,

(13) $$GE(G\left(V^{c},E^{c})\right)=\overset{N}{\underset{i=1}{\sum }}\lambda_{i}\left(\left[E^{c}\right]\right).$$

Since *GE *depends only on the adjacency matrix, the density of the graph does not affect its computation time. The effect of query interaction on the original graph structure is captured by change in *GE *(i.e. Δ*GE*(*c, t*)), given similarly like equation (8). We are dealing with graphs whose edge strength is the similarity between connecting nodes, which is inverse of the usual edge length definition. So we need to look for the maximum Δ*GE*(*c, t*).

### Algorithm

Graphs are analyzed hierarchically (as discussed in section 'Graph analysis') and threshold plays an important role in making hierarchical graph structure. If the query can interact at the higher layer of GP (see Figure 2(a)), then it means it is a strong interaction and it accounts for the global feature. Because in GP as the level rises, the threshold also increases and at every level, the graph edges can only be formed if their strength is greater than the given threshold. Here interactions are in the sequence similarity space, so a sstrong interaction means the highest similarity between corresponding sequences, which occurs when both sequences match at most of the nucleic acids (i.e. match globally). Hence strong interaction accounts for the global feature and similarly weak interactions account for local features.

**Figure 4:**
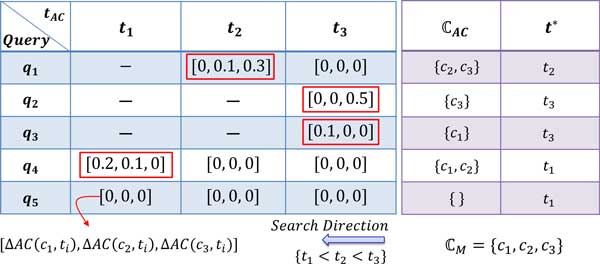
**Illustration of the graph pyramid search subroutine**. Here we elaborate the algorithm 1 using 5 different queries. For simplicity assume *T_AC _*= 0, *t_AC _*= {*t*_1_*, t*_2_*, t*_3_} and only 3 protein classes i.e. $|{\mathbb{C}}_{M}|=3$. Each query *q_i_*, is analyzed first at the highest level (*t*_3_) where we look for all classes $c\in {\mathbb{C}}_{M}$, having Δ*AC*(*c, t*_3_) *> T_AC _*and collect them in ${\mathbb{C}}_{AC}\subseteq {\mathbb{C}}_{M}$. For *q*_1 _we can not find any such classes *c *at *t*_3_, so we descend the GP to *t*_2 _level and discover ${\mathbb{C}}_{AC}=\left\{c_{2},c_{3}\right\}$ with threshold *t^∗ ^*= *t*_2_. This secondary threshold (*T_AC_*) is necessary, otherwise there will be many spurious classes (false positives (FP)) having nonzero Δ*AC*(*·*).

Let $T=\{\left\{t_{AC}\right\},\left\{t_{RC}\right\},\left\{t_{SM}\right\},\left\{t_{TR}\right\},\left\{t_{GE}\}\right\}$, be a set of sets, defining few threshold levels corresponding to each GTF. Consider, *t_AC _*= {*t*_1_*, t*_2_*, t*_3_} and *t*_1 _*< t*_2 _*< t*_3_. Let us consider there are only 3 protein classes i.e. $\left|{\mathbb{C}}_{M}\right|=3$. Figure 4 explains the hierarchical query classification subroutine (algorithm 1) for the feature *AC*. Each *q_i _*is analyzed first at the highest level (*t*_3_) where we look for class $c\in {\mathbb{C}}_{M}$, having Δ*AC*(*c, t*) *> T_AC _*and collect them in ${\mathbb{C}}_{AC}\subseteq {\mathbb{C}}_{M}$. For query *q*_1 _we can not find any such classes *c *at *t*_3_, so we descend the GP to *t*_2 _and discover ${\mathbb{C}}_{AC}=\left\{c_{2},c_{3}\right\}$ with threshold *t^∗ ^*= *t*^2^. The secondary threshold (*T_AC_*) is necessary, otherwise there will be many spurious classes i.e. false positives (FP), having nonzero change in average clustering coefficient (ΔAC(⋅)≠0).

In the subroutine given in an algorithm 1, *t^∗ ^*is the maximum threshold (also defines maximum GP level) at which the query starts interacting with the graph of some class. Let us represent the subroutine by abusing the notation for simplicity and readability as,

(14) $${\mathbb{C}}_{AC}\leftarrow \underset{c\in {\mathbb{C}}_{M}}{\text{argmax}}\;H\left(\text{Δ}AC\left(c,t^{*}\right)-T_{AC}\right).$$

Maximum value of *H*(*·*) is 1, so all the arguments (classes *c*) are assigned to ${\mathbb{C}}_{AC}$ whenever it produces output 1. For reducing FP, the subroutine for *SM *and *TR *slightly changes (see algorithm 2). Here we look for *k *maximally influenced classes from ${\mathbb{C}}_{RC}$ by the query. Thus classes from only ${\mathbb{C}}_{RC}$ are assessed (voted) again by the features *SM *and *TR*. Subroutine for *GE *finds the class from ${\mathbb{C}}_{SM}\cup {\mathbb{C}}_{TR}$ for which Δ*GE*(*·*) is maximum. Thus this produces a hierarchical class voting scheme which helps to improve the classification accuracy and to reduce the computational load.

Each GSF has an ability to extract different information from different levels of the protein family GP. However, applying each GSF to entire GP, is computationally inefficient when dealing with large numbers of protein families. In addition, it may add up the FP, when a decision is being made in the much lower GP level than the level defined by *t^∗^*. To avoid these problems, the GP based hierarchical voting scheme is necessary. And the rational behind placing different GSF at different GP levels, is explained in the next 'Results' section.

**Algorithm 1 **Graph pyramid search subroutine

input: $s_{q}^{c_{q}}$; secondary threshold *T_AC_*; primary threshold $t_{AC}=\left\{t_{1},t_{2},\cdots \;,t_{n}\right\}$, where *t_i _*>*t_j _*for *i *>*j*

output: ${\mathbb{C}}_{AC},t^{*}$

1: ${\mathbb{C}}_{AC}=\emptyset$

2: **for ***t *∈ *t_AC _*from *t_n _*to *t*_1 _**do**

3:   **if **${\mathbb{C}}_{AC}=\emptyset$ **then**

4:      **for all **classes $c\in {\mathbb{C}}_{M}$**do**

5:         *I_AC _*= *H *(Δ*AC*(*c,t*)−(*T_AC_*))

6:         ${\mathbb{C}}_{AC}={\mathbb{C}}_{AC}⊎\delta \left(c,I_{AC}\right)$

7:         t* = *t*

8:      **end for**

9:   **end if**

10: **end for**

## Results

### Dataset and evaluation details

Proposed method is evaluated on entire COG database [6]. It is the protein database of Clusters of Orthologous Groups (COG). It consists of 4,873 COG (protein families), having total 138,458 proteins from 66 different genomes. Approximately 10% sequences from each COG, are selected randomly, which has produced 14,086 test sequences. This procedure is repeated 5 times further to get average performance. First, each GSF is tested independently, for various thresholds without using hierarchical voting scheme. Here, the class which produces maximum change in the GSF for a given $s_{q}^{c_{q}}$, is selected as the output. These output labels are produced with either correct decision (*cd*), wrong decision (*wd*) or no decision (*nd*, when $|c_{q}|\neq 1$). Then let the performance measure be defined as, $precision=\frac{cd}{cd+wd}$.

### Rational behind hierarchical voting

**Figure 5:**
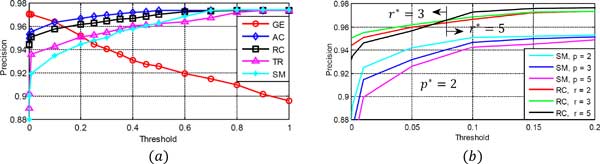
**Performance analysis of each GSF and their parameters**. (*a*) Performance of all the GSF at the various thresholds (GP levels); (*b*) Optimal parameters search for the *SM *and *RC*. For a given particular threshold, the parameters producing high precision are selected after the cross validation for training the model.

**Figure 6:**
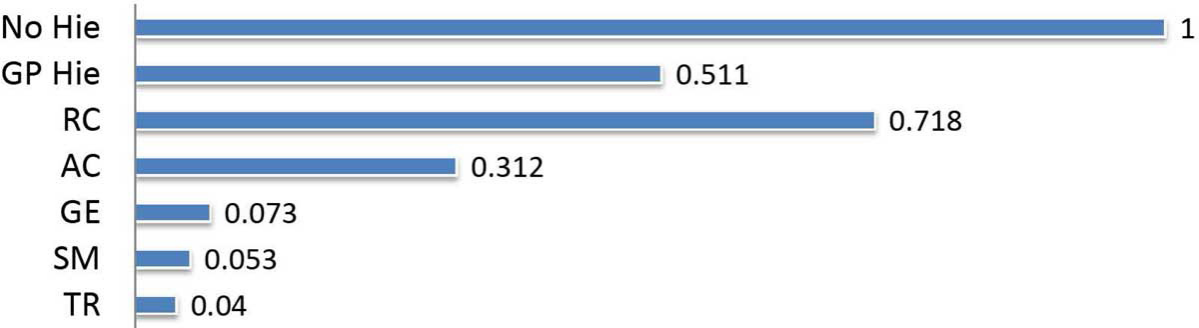
**Normalized testing time for different classifiers**. 'GP Hie' is the proposed graph pyramid hierarchical implementation and 'No Hie' is the majority voting of GSF without using the hierarchical procedure.

Figure 5(a) shows the plots of precision Vs threshold for all GSF. High precision indicates low wrong decisions. *AC *and *RC *produce high precision as the threshold rises, so these GSF are appointed to work at higher levels of GP. So at a high threshold, it is more likely that ${\mathbb{C}}_{AC}$ and ${\mathbb{C}}_{RC}$ contain true output class. Thus it is sufficient to apply other GSF, to the GP generated from either ${\mathbb{C}}_{AC}$ or ${\mathbb{C}}_{RC}$. On the other hand, *GE *produces high precision for low thresholds. One of the possible reasons behind this is that, the higher the threshold, the sparser will be the graph. So eigen-decomposition of the adjacency matrix to calculate *GE *will not give any information as Δ*GE *is close to zero for all classes. While at low threshold, the original graph becomes dense and query also interacts with almost all nodes in the true output class graph. Immensity in the interaction at lower threshold, helps *GE *to detect the true class easily and correctly. This forces *GE *to work at lower levels of GP, with the smallest search space as ${\mathbb{C}}_{SM}\cup {\mathbb{C}}_{TR}$. With similar arguments, *SM *and *T R *are placed at intermediate GP levels, allowing them to look for *c_q _*in ${\mathbb{C}}_{RC}$. So in the algorithm 2, hierarchy as well as input search space for GSF are organized carefully. This helps to reduce the search space dramatically for other GSF and thus speeds up the algorithm (Figure 6 for speed comparison).

**Algorithm 2 **Classification by hierarchical voting

training: all G(V^c^,E^c^) are constructed $\forall c\in {\mathbb{C}}_{M}$

input: $s_{q}^{c_{q}},r,p,k$, primary threshold set  T, secondary thresholds *T_AC_*, *T_RC_*

**output: ***c_q_*

1: ${\mathbb{C}}_{AC}\leftarrow \underset{c\in {\mathbb{C}}_{M}}{\text{arg max}}\;H\left(\text{Δ}AC\left(c,t^{*}\right)-T_{AC}\right)$

2:${\mathbb{C}}_{RC}\leftarrow \underset{c\in {\mathbb{C}}_{AC}}{\text{arg max}}\;H\left(\text{Δ}RC\left(c,t^{*},r\right)-T_{RC}\right)$

3: ${\mathbb{C}}_{SM}\leftarrow \underset{c\in {\mathbb{C}}_{RC}}{\text{arg k-max}}\text{ Δ}SM\left(c,t^{*},p\right)$

4: ${\mathbb{C}}_{TR}\leftarrow \underset{c\in {\mathbb{C}}_{RC}}{\text{arg k-max}}\text{ Δ}TR\left(c,t^{*}\right)$

5: ${\mathbb{C}}_{GE}\leftarrow \underset{c\in {\mathbb{C}}_{SM}\cup {\mathbb{C}}_{TR}}{\text{arg k-max}}\text{Δ}GE\left(c,t^{*}\right)$

6: \∗ *get the class label having the highest frequency* ∗\

7: *ψ_q _*= mode $\left({\mathbb{C}}_{AC}⊎{\mathbb{C}}_{RC}⊎{\mathbb{C}}_{SM}⊎{\mathbb{C}}_{TR}⊎{\mathbb{C}}_{GE}\right)$

8: \∗ *resolve the indecisive case step by step* ∗\

9: **if ***|ψ_q_| ≥ *2 **then**

10:   ${\mathbb{C}}_{SM}\leftarrow \underset{c\in {\mathbb{C}}_{RC}}{\text{arg max}}\text{ Δ}SM\left(c,t^{*}\right)$

11:   ${\mathbb{C}}_{TR}\leftarrow \underset{c\in {\mathbb{C}}_{RC}}{\text{arg max}}\text{ Δ}TR\left(c,t^{*}\right)$

12:   $\psi_{q}^{\prime }=\text{mode(}{\mathbb{C}}_{SM}⊎{\mathbb{C}}_{TR}⊎{\mathbb{C}}_{GE}\text{)}$

13:    **if $\psi_{q}^{\prime }\geq 2$ then**

14:      $c_{q}={\mathbb{C}}_{GE}$

15:   **else**

16:      $c_{q}=\psi_{q}^{\prime }$

17:   **end if**

18: **else**

19:   *c_q _*= *ψ_q_*

20: **end if**

Sometimes, for the query $s_{q}^{c_{q}}$, having subtle interactions with many classes, it is difficult for all GSF to come up with an unique agreement about *c_q _*. When it happens, the threshold would have already hit the bottom of its range. Thus, with earlier reasoning, the solution would be to rely only on *GE *to find *c_q _*, and 14*^th ^*step in the algorithm 2 does the same.

### Deciding algorithmic parameters

Figure 5(b) shows the precision Vs threshold plots for *SM *and *RC *with different parameters. This helps to decide optimal parameters (*p^∗^, r^∗^*) for them at the particular threshold. In the implementation, *p^∗^*= 2 for all GP levels, while *r^∗^* is set 3 for lower and 5 for higher GP levels. Other thresholds are set using the validation set by analyzing the maximum values for each GSF. And each set in the T has the uniformly quantized numbers from 0 to maximum GSF value.

### Performance analysis

Figure 6 shows the normalized time taken by different classifiers for testing 14,086 sequences. In Majority voting scheme, first all GSF classify each sequence from the large pool of testing sequences, and then the voting begins. This slows down the scheme. On the other hand, in the proposed GP hierarchical scheme, the testing pool is gradually shrunken down. So the subsequent GSF have to investigate only small set of sequences, which likely to contain the true protein class. Which in turn speeds up the proposed algorithm along with maintaining high accuracy.

**Figure 7:**
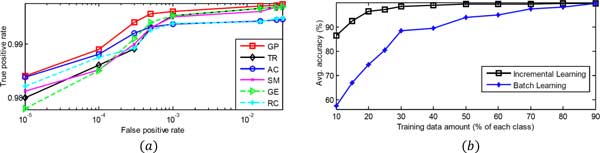
**Performance evaluation and the incremental learning**. (*a*) ROC curves for individual GSF (independently evaluated) and their combination in GP using majority voting; (*b*) Performance comparison of the Incremental and the Batch learning.

GP based modeling of protein families provides an extra advantage of fast incremental learning. In this procedure, $s_{q}^{c_{q}}$ is added back to cq after its classification. This is an instance based learning and it only requires slight modification to the graph of protein family *c_q_*, like replacing *G*(*V^c_q_^, E^c_q_^*) with $G\left(V_{q}^{c_{q}},E_{q}^{c_{q}}\right)$. Experiment consists of randomly selecting various (10 to 90) percent data from each $c\in {\mathbb{C}}_{M}$ for training purpose. This procedure is repeated 5 times and the Figure 7(b) shows the average classification performance comparison between incremental and batch learning. For small amounts of training data, batch learning performs poorly. On the other hand, incremental learning takes advantage of each correctly classified $s_{q}^{c_{q}}$ and produces better performance even during the scarcity of the training data.

Improvement in the classification performance can be achieved by combining the GSF in GP and using majority voting. This is shown more elaborately with ROC curves in Figure 7(a). For all FP rates, GP (GSF combination) always produces better true positive rate than individual GSF. In '*mean *bit score' classification method, bit score is used as an edge weight for constructing the graph. The class, $c\in {\mathbb{C}}_{M}$, which produces the maximum mean of the weights of newly formed edges by $s_{q}^{c_{q}}$, is decided as *c_q_*. The performance of this type of bit score based methods, is poorer than EB-score based methods. Top part of the Table 1, shows the importance of the EB-score and the GP; and bottom part of it compares the performance of the proposed method with various methods. When the GSF are not used hierarchically with the GP, they fail to extract enough information from protein family for classification. Thus the accuracy of 'TR without GP' is only 90%, while that of 'TR with GP hierarchy' is 98.9%. The proposed majority voting scheme without hierarchy (No Hie), shows better accuracy than the Tree-kNN based state of the art method [27], while GP based hierarchical voting (GP Hie) scheme produces the best accuracy. Being twice faster than 'No Hie', with maintaining high performance, the 'GP Hie' method is the preferable solution for a time consuming protein classification task.

**Table 1:** Average number of protein sequences misclassified (out of 14,086 testing sequences from COG [6] database) by the different protein classification methods.

*max *bit-score	362.3	*max *EB-score	263.2
*sum *bit-score	587.5	*sum *EB-score	238.1
*mean *bit-score	970.0	*mean *EB-score	308.4
*T R *without GP	1404	*T R *with GP hierarchy	150.7
KNN classifier			309.7
Highest scoring BLAST match [2]			362.9
Boujenfa et al. (using ClustalW) [27]			35.2
Proposed majority voting of GSF (No Hie)			28.2
Proposed GP based hierarchical voting (GP Hie)			**21.1**

## Discussion and conclusions

As discussed initially in the background section, here we took an approach based on protein homology for protein function prediction. According to this approach an entire task boils down to protein classification, because two proteins with similar sequence or structure could evolve from a common ancestor and thus have similar functions. So once we classify a protein to its true family, we can easily ascertain its probable functions from the characteristics of its family. We took this approach because it is fast, an approximate and primary way to tackle a daunting task of function prediction of a large number of proteins.

This paper proposes a novel protein classification method based on PPS network modeling using the proposed EB-scores. It tries to blend important characteristics from PPI network and ISS methods for protein classification. Importance of the method is that it exploits the topological structural information of the PPS network, using hierarchical network analysis guided by the graph pyramid. This helps to analyze the different protein interactions at different pyramid levels. Thus the necessary information for protein classification from weak interactions in the PPS network is not suppressed by the other strong interactions. And proposed features extract the different network properties at various pyramid levels. This makes it possible to more objectively and reasonably predict the protein class.

The hierarchical voting algorithm helps to improve the computational efficiency with maintaining high classification accuracy. Some of the salient features of the proposed method are; protein sequences as the only input requirement; fast and easy incremental learning; can show topologically, how the query sequence interacts with the protein family; quick training and the high performance. The proposed graph based modeling, has an extra advantage that the relationship between protein families can also be found by finding the corresponding inter-graph similarities. The experimental evaluation on COG database demonstrated the effectiveness of the proposed method.

This graph pyramid approach is also promising to use in the PPI network and various other graph based bioinformatics methods. Protein characteristics like 3D structure and presence of various domains, along with sequence similarity measure can be used for more efficient protein network construction. Our future work will try to address these issues.

## Competing interests

The authors declare that they have no competing interests.

## Authors' contributions

All authors read and approved the final manuscript. TS proposed the algorithm and drafted the paper. YJ helped for dataset analysis. KS proposed, supervised the project, gave suggestions and revised the manuscript. JYC gave suggestions and revised the manuscript.
